# Models to reconcile plant science and stochasticity

**DOI:** 10.3389/fpls.2014.00643

**Published:** 2014-11-14

**Authors:** Sam Collaudin, Vincent Mirabet

**Affiliations:** ^1^Reproduction et Développement des Plantes, INRA, CNRS, Ecole Normale Supérieure de Lyon, Université Claude Bernard Lyon 1Lyon, France; ^2^Laboratoire Joliot-Curie, CNRS, Ecole Normale Supérieure de LyonLyon, France

**Keywords:** stochasticity, plant development, modeling, pattern, morphogenesis, tissue

Plants are modular organisms that exhibit diverse adaptations to variability. This variability can be intrinsic in nature, as in the case of cell shape or division plane stochasticity, protein distribution in a cell, variations in internal mechanical properties etc… (Altschuler et al., 2008; Besson and Dumais, 2011). It can also be extrinsic, as with variations in environmental conditions at different time scales (Wolpert et al., 1998; Sultan, 2000; Franklin, 2009; Leyser and Day, 2009). When it comes to rationalizing data acquisition and interpretation, one has the tendency to define what part of the variability is arguably unhelpful stochasticity and what part does in fact contain meaningful information.

Systems biology, which combines methodologies from various disciplines, can be used to understand the mechanisms of development. For example, complex network analysis (Lucas et al., 2011), computer simulations (Band et al., 2012) or physical measurements through atomic force microscopy (Milani et al., 2014) can be combined with biological experiments. For instance, such an approach has been able to produce reasonable explanations for how patterning at the meristem level can lead to the stem structure (Prusinkiewicz et al., 1995). Stochasticity in models as a variable or as a methodological tool has been a subject of interest for many years in physics and mathematics (Saguès et al., 2007; Friedrich et al., 2011; Wilkinson, 2011). Studies have already been published in biology but only a few focused on plant development, and are often more recent (for a review of this aspect, see Meyer and Roeder, 2014). Along with a better understanding of growth processes, those studies have also illustrated how our vision of stochasticity was previously too derogatory (Kliebenstein, 2012). Those new methodologies illustrate how stochasticity can be both a consequence and an origin of core mechanisms in development.

Here we use specific examples to illustrate how mathematical or computational models are well-suited to the study of stochasticity in plant functions. Moreover, models enable the use of measured phenotypic stochasticity at multiple scales to elucidate the underlying processes. We suggest that models used for such purposes do not need to be overly complex, and various complex models of the same process will in fact converge toward similar conclusions. We will focus our attention on apical meristems and the growth that they generate, where cell–cell interactions underlie the emergence of various interesting properties of the tissues and organs.

## Stochasticity can be buffered by genetic networks at cellular and tissular scales

It has been shown that low levels of a protein or a chemical component induce a high level of noise that can impact on pattern formation (Shnerb et al., 2000). For instance, in *Drosophila*, the *Hunchback* (*Hb*) gene is crucial for the proper segmentation of the embryo, and is regulated by *Bicoid* (*Bcd*). The Bcd gradient can cause intense noise due to the small numbers of molecules. Nevertheless, the definition of segmentation and boundary position are well-conserved between embryos despite Bcd stochasticity. Holloway proposed in his study that noise can be affected by the number and the strength of binding sites on a promoter (Holloway et al., 2011). He develops computational models to confirm that the high number of Bcd binding sites on the Hb promoter reduce stochastic noise of Bcd gradient.

Complex gene regulatory networks drive morphogenesis in all multicellular contexts. Feedback and redundancy within these networks compensate for intrinsic or extrinsic stochasticity. For instance flower formation is driven by the activation of a small network mainly composed by *LEAFY (LFY)*, *APETALA1 (AP1)*, *CAULIFLOWER (CAL)*, and *TERMINAL FLOWER1 (TFL1)*. This network is regulated by environmental and physiological inputs to start flower initiation at the appropriate time for reproduction. This network contains many feedback loops and mutual activations, such as the induction by LFY of AP1 and CAL, which themselves positively regulate LFY. These interactions can buffer the environmental noise to obtain the formation of a robust pattern and to avoid the reversal of flowering (Blazquez et al., 2006).

## Models explain the robustness of patterning

Plant tissues, even those as little differentiated as meristems, exhibit strong self-organizational properties. Intense local cell–cell interactions through diverse exchanges (Murray et al., 2012; Landrein and Vernoux, 2014) contribute to the emergence of patterns. Those patterns may be in the form of either simple genetic differentiation or more complex morphogenetic events. Among the various properties of such self-organized patterning, robustness is crucial for the principal meristematic properties: their ability for self-renewal or to produce various lateral organs. Models are well-suited to predict how a set of linked cells can generate shape and differentiate following emergent processes.

Auxin signaling processes are amongst the best-studied cases of tissue patterning in plants, and furthermore, they have also been extensively modeled and linked to numerous biological observations. In the auxin flux models, simple cell-to-cell communication occurs via the local amplification of auxin flux by the PIN-FORMED proteins. The auxin response patterns observed in the meristematic tissues of the stem and at the vascular generation zones, are an emergent property of those molecular interactions (Sassi and Vernoux, 2013). This system clearly illustrates the robustness of emergent patterns to external noise. External noise can be as intense as multicellular injuries. It has been shown that an injury to the meristem can be compensated. The patterning system is robust enough and can maintain the activity of organogenesis (Snow and Snow, 1932; Reinhardt et al., 2005). Computational Models of phyllotaxis can predict how the plant might cope with such ablations; the pattern is very quickly deformed around the ablation, but re-emerges naturally as growth continues to produce healthy new cells. This resistance to local injuries is also observed in vasculature development. Cutting a part of the provasculature induces its spontaneous reconfiguration, such that the new vasculature is reshaped around the ablation (Sauer et al., 2006). Models predict that such reconfiguration does not need any specific change in cell behavior, and that cell–cell communication itself is sufficient to enable such changes (Wabnik et al., 2010).

## Stochasticity in patterning is filtered in plant tissues

If spatial robustness is a natural outcome of the self-organization described above, this kind of patterning is itself sometimes stochastic. The phyllotactic angle between successive lateral organs forming on the shoot apical meristem (SAM) is approximately 137 degrees in *Arabidopsis thaliana*. For a long time, research has focused on predicting mechanisms behind the astonishing regularity of phyllotaxis in various plant systems (Adler, 1997). More recently, the close examination of plants phyllotaxis has led to the discovery of strange phenotypic alterations. The *histidine phosphotransfer protein 6 (ahp6)* mutant presents curious alterations called M-shaped successions, where three successive lateral organs display altered angles (Besnard et al., 2013). Other, more complex, successions are visible with lower frequencies. Strikingly, these types of alterations also occur in wild type plants, though less frequently. In order to understand the source of this stochasticity and the specific pattern of alterations, both statistical (Refahi et al., 2011; Guédon et al., 2013) and agent (Mirabet et al., 2012) models have been used to study phyllotaxis in *Arabidopsis*. These models have predicted that the auxin system, under the influence of stochasticity, can spontaneously generate the alterations seen along the stem. Indeed in mutants some organs can be generated simultaneously, whereas a delay (called the plastochron) occurs in a typical normal situation. Because the organs appear simultaneously, the way they are arranged along the stem may be inverted, thus producing the characteristic M-shaped structure. This stochasticity in timing would thus appear to be a spontaneous outcome of the spatial patterning of the auxin system.

These studies have helped clarify that the *Arabidopsis* SAM possesses a second patterning system, based on the AHP6 protein, that partially overlaps the auxin system and ensures that new primordia will emerge successively through time. Thus, the temporal stochasticity of the auxin system is compensated for by a second patterning process that filters it. Without the use of a “systemic” view of the entire patterning process, it would have been difficult to decipher the role of the AHP6 system.

## Stochasticity as a source of patterning and morphogenesis

In developmental biology, stochastic gene expression can lead to the formation of coherent patterns. An example is in the ommatidium of the *Drosophila* eye, which consists of eight photoreceptor cells. Two of them (R7 and R8) express rhodopsin, which is responsible for the detection of color. It has been shown that the separation of “yellow” and “pale” ommatidia determined by rhodopsin regulation in R7 and R8 is due to the stochastic expression of the *SPINELESS* receptor (Wernet et al., 2006). This stochasticity is both necessary and sufficient for proper ommatidial development. In this example, stochastic gene expression at the cell level can become instructional at the tissue level.

Through the use of simple activator-inhibitor model systems, Turing managed to describe the self-organization of various spatial patterns (Turing, 1952). These patterns mainly depend on the strength of molecular interactions and on the geometry of the domains where the activators and inhibitors are expressed. In these computational models, stochasticity is necessary to trigger the dynamics that leads to the final stable pattern. Stochasticity of cell behaviors becomes the motor of patterning. Nevertheless, this stochasticity is in a way buffered by the interactions, as its intensity has only a small effect on the final pattern. In plants, examples of such systems exist in trichomes positioning in leaves (Benítez et al., 2007; Greese et al., 2012). Interactions can be summarized into an activator complex (that consists of *WEREWOLF*, *GLABRA1*, *GLABRA3*, *ENHANCER OF GLABRA3*, and *TRANSPARENT TESTA GLABRA*) as well as the redundant inhibitory activity of *CAPRICE* and *TRIPTYCHON*. With Turing-like models applied to those components, the authors were able to reproduce the experimentally observed patterns.

Stochasticity is present not only in gene expression, but is an inherent property of cells, notably with respect to cell growth. A recent study showed that cells are able to interact mechanically to adapt their growth depending on the behaviors of their neighbors (Uyttewaal et al., 2012). Interestingly, this function seems to increase variability instead of compensating for it. In turn this positive feedback is necessary for correct morphogenesis of new primordia. Models predict that an optimum exists between variability of cell growth and feedback between cells. Depending upon the relative strength of both parameters, the tissue can grow more or less efficiently. This intricate interplay between stochasticity and cell–cell communication is a fundamental aspect of tissue morphogenesis, and would appear to be regulated. Models can help predict the optimal ratio between stochasticity and feedback necessary for proper morphogenesis. Interestingly, it is not this theoretical optimum that seems to be generated in meristems, a fact that may allow the tissue to undergo growth bursts, which may in turn lead to primordia emergence (Alim et al., 2012).

## Simple models translate variable phenotypes into valuable information

Complex systems can be modeled quite simply. An example is human crowds being modeled as simple interacting agents with very basic properties. Such models can efficiently predict the behavior of these groups (Helbing et al., 2000). Similarly, plant cells and tissues can also be modeled using such approaches. With a simple model such as that of Turing (with less than 10 parameters), it is possible to add noise measured at the cell scale, and study its consequences at an higher (tissue or plant) level. Thus, phenotypic variability at this higher level can be interpreted through the model, that gives the ability to search for the cellular parameters leading to the mutant phenotype of interest.

In the example of phyllotaxis described above, the types and frequencies of alterations may be interpreted through the use of the model. They are predicted to be an outcome either of alterations of the meristem structure or the auxin system. This scenario may be easily tested with further experimentation, for example searching for defects in the pin network or meristematic size.

This reasoning is in fact multiscale, each conclusion at one scale providing the data for models that focus on the link to the next level of organization. Our ability to measure variability and stochasticity at various scales has recently been increased. Experimental techniques and analysis tools have immensely improved the precision of measurements both spatially and temporally, and at various scales (Fernandez et al., 2010; de Reuille et al., 2014). Those new techniques point out the importance of heterogeneity and stochasticity in biological systems. Modeling approaches will be more and more helpful in this new context to explain those data.

It is time to switch from seeing biology as clockwork perfection to looking at its natural variations more thoroughly. That will undoubtedly help us decipher where plants' real beauty is hidden: behind those so-called imperfections.

### Conflict of interest statement

The authors declare that the research was conducted in the absence of any commercial or financial relationships that could be construed as a potential conflict of interest.
